# Evaluating Patient-Centered Mobile Health Technologies: Definitions, Methodologies, and Outcomes

**DOI:** 10.2196/17577

**Published:** 2020-11-11

**Authors:** Courtenay Bruce, Patricia Harrison, Charlie Giammattei, Shetal-Nicholas Desai, Joshua R Sol, Stephen Jones, Roberta Schwartz

**Affiliations:** 1 System Quality & Patient Safety Houston Methodist System Houston, TX United States; 2 CareSense MedTrak, Inc Conshohocken, PA United States; 3 Center for Innovation Houston Methodist Hospital Houston, TX United States; 4 Information Technology Division Houston Methodist Hospital Houston, TX United States; 5 Center for Outcomes Research Houston Methodist Research Institute Houston, TX United States; 6 Department of Surgery Houston Methodist Hospital Houston, TX United States

**Keywords:** innovation, health care, digital technology, digital interventions, patient-facing technologies, patient-centered care, patient centeredness, patient experience, patient engagement, patient activation, quality, effectiveness, quality improvement, information technologies, outcomes, readmissions, length of stay, patient adherence

## Abstract

Several recently published studies and consensus statements have demonstrated that there is only modest (and in many cases, low-quality) evidence that mobile health (mHealth) can improve patient clinical outcomes such as the length of stay or reduction of readmissions. There is also uncertainty as to whether mHealth can improve patient-centered outcomes such as patient engagement or patient satisfaction. One principal challenge behind the “effectiveness” research in this field is a lack of common understanding about what it means to be effective in the digital space (ie, what should constitute a relevant outcome and how best to measure it). In this viewpoint, we call for interdisciplinary, conceptual clarity on the definitions, methodologies, and patient-centered outcomes frequently used in mHealth research. To formulate our recommendations, we used a snowballing approach to identify relevant definitions, outcomes, and methodologies related to mHealth. To begin, we drew heavily upon previously published detailed frameworks that enumerate definitions and measurements of engagement. We built upon these frameworks by extracting other relevant measures of patient-centered care, such as patient satisfaction, patient experience, and patient activation. We describe several definitional inconsistencies for key constructs in the mHealth literature. In an effort to achieve clarity, we tease apart several patient-centered care outcomes, and outline methodologies appropriate to measure each of these patient-care outcomes. By creating a common pathway linking definitions with outcomes and methodologies, we provide a possible interdisciplinary approach to evaluating mHealth technologies. With the broader goal of creating an interdisciplinary approach, we also provide several recommendations that we believe can advance mHealth research and implementation.

## Background

Mobile health (mHealth) is defined by the Word Health Organization as “medical and public health practice supported by mobile devices, such as mobile phones, patient monitoring devices, personal digital assistance, and other wireless devices” [1,2]. mHealth is considered the future of health care [3,4], and many health care organizations have embraced mHealth as part of their patient-centered initiatives. Specifically, according to a US News & World report, 18 of the top 20 medical centers have adopted and widely celebrated mHealth technologies, at least in a review we conducted of their websites [5]. The National Institutes of Health funding for developing, testing, and implementing mHealth interventions grew from US $16.8 million in 2014 to US $39.4 million in 2018 [6].

Despite the growth and hype of mHealth technologies, there is a paucity of effectiveness evidence in the literature to support such widespread implementation [6]. Several recently published studies and consensus statements have demonstrated that there is only modest (and in many cases, low-quality) evidence that mHealth can improve patient clinical outcomes such as lengths of stay or reduction of readmissions [6-9]. There is also uncertainty as to whether mHealth can improve patient-centered outcomes such as patient engagement or patient satisfaction [6-10]. One principal challenge behind the “effectiveness” research is a lack of shared understanding about what it means to be effective in the digital space (ie, what should constitute a relevant outcome and how best to measure it) [2]. This lack of a shared understanding can likely be attributed to the multidisciplinary, multifaceted nature of mHealth, often involving disciplines such as engineering, data science, systems science, human-computer interaction, behavioral sciences, public health, and medicine [10-13]. Each discipline tends to frame its effectiveness research in terms of its own specialized knowledge [13], thereby limiting the generalizability of research results across domains.

In this viewpoint, we call for interdisciplinary, conceptual clarity on the definitions, methodologies, and patient-centered outcomes frequently used in mHealth research. There have been recent, important calls in the literature for conceptual clarity about engagement [6,10-13]; however, as we demonstrate below, engagement is only one facet of patient-centered care [14] and it is not always clear that sustained engagement is required to achieve mHealth outcomes [2]. Stated differently, a shared understanding about engagement is necessary but not sufficient for an interdisciplinary research approach. Conceptual clarity on engagement can only go so far to reduce the current fragmentation of research efforts [12]. Instead, what is needed is consideration beyond one measure of patient-centered care to include several measures of patient-centered care and the methods for evaluating them. By creating a common pathway linking definitions with outcomes and methodologies, we hope to draw a comprehensive (but not necessarily exhaustive) outline of a possible interdisciplinary approach to evaluating mHealth technologies.

To formulate our recommendations, we used a snowballing approach to identify relevant definitions, outcomes, and methodologies related to mHealth. To begin, we drew heavily upon the work of Perski et al [12], Yardley et al [6,13], and Short et al [10], among others [14,15], whose detailed frameworks enumerate the definitions and measurements of engagement [15]. We built upon their frameworks by extracting other relevant measures of patient-centered care, such as patient satisfaction, patient experience, and patient activation, drawing on the quality and patient safety literature—a literature base that has, thus far, to our knowledge, been largely untapped in the mHealth context.

Quality health care refers to care that is safe, effective, patient-centered, timely, efficient, and equitable [11,16]. In the mHealth context, most of the work comes from informaticians rather than health care quality practitioners. Theory-based informatics research must be informed by the frontline real-world, hospital environment where health care quality takes place, because patients are different than consumers or research participants [11,17]. Patients experience a constellation of complex, emotionally laden perspectives during their use of mHealth technology that may not be considered when conducting informatics research outside of the hospital setting [18]. The field of health care quality can help advance mHealth research in its evidence-based emphasis on the patient, his or her experiences, and how the continuum of care can influence outcomes that matter to the patient [19,20]. 

A data extraction table was used to sort, explore, and synthesize existing research (see Multimedia Appendix 1). We filtered and coded our findings based on whether a definition was proposed, whether outcome measures were discussed (and what the outcome measures were), and methodologies used to assess the outcomes. We reran the search queries that were performed in 5 recent, frequently cited systematic reviews [6,12,14,21,22] using the Medline, PsycINFO, PubMed, and Google Scholar databases. Although we did not intend to perform a systematic review for this Viewpoint, we believe that this research approach was comprehensive and could be used to formulate recommendations based on gaps and inconsistencies in the literature.

## Defining Patient-Centered mHealth Technologies

A survey of the literature highlights the myriad terms used in mHealth and the various ways in which they are defined. The terms “mHealth,” “telehealth,” “eHealth,” and “digital technologies” are often treated synonymously [23], although, in reality, they could be different in that eHealth or digital technologies can encompass devices that are not supported by mobile means, such as hospital check-in or registration portals to more advanced technologies designed to enhance patient understanding through education and communication such as Smart Boards and Smart TVs located on hospital units [24].

Digital behavior change interventions (DBCIs) are a subset of eHealth, defined as “a product or service that uses computer technology to promote behavior change,” which can be delivered through computer programs, websites, mobile phones as text message, smartphone apps, or wearable devices [6].

Patient-centered care is a health care quality indicator proposed by the Institute of Medicine (IOM) [25]. Patient-centered care has generally been poorly defined, with authors often conflating several distinct concepts such as using the term “patient-centered care” when they are usually referring to a specific outcome measure of patient-centered care [26]. As the IOM explains, patient-centered care is care that is “respectful of and responsive to individual patient preferences, needs, and values, and ensuring that patient values guide all clinical decisions” [25]. Through this lens, patient-centered care can be conceptualized as an overarching, broad concept that puts emphasis on respecting patients as a means of honoring their dignity and worth [26,27]. By extension, patient-centered mHealth technologies can be defined as technologies supported by mobile devices designed to promote patient-centered care. There are several outcome measures for patient-centered care.

## Patient-Centered Care Outcomes for Patient-Centered mHealth Technologies

“Patient experience” is likely the strongest, most proximate outcome measure of patient-centered care. Patient experience refers to any process observable by patients, including subjective experiences (eg, pain control) and their objective experiences (eg, wait time) [28,29]. A primary feature of patient experience is that it reflects actual health care experiences [30]. Another important feature of patient experience is that it refers to a time sequence. Specifically, patient experience can refer to the first touchpoint within an episode of care (eg, being assessed for a left knee replacement) to the last touchpoint within that episode (eg, last follow-up appointment following surgery for a left knee replacement). Alternatively, patient experience can refer to the whole continuum of care—their first encounter with Hospital A to their last encounter with Hospital A.

“Patient engagement” and “patient activation” are two outcome measures of patient-centered care that are distinct from patient experience. In particular, “patient engagement” has been defined in various ways in the literature [14,30,31]. Recent proposals for integrative definitions of engagement have defined engagement as consisting of objective and subjective components [6,12,13]. The objective component is the extent (eg, amount, frequency, duration, and depth) of usage of the mHealth technology [12]. With respect to what constitutes sufficient objective engagement, the literature demonstrates a palpable lack of consensus [15,31]. Some researchers have proposed that a certain empirical threshold of engagement must be met to show sufficient engagement with the intervention to achieve intended outcomes [13], whereas others suggest that one critical point of engagement or fluid, ebbing, and flowing engagement may be sufficient [32,33]. The subjective element of engagement is often characterized by the user’s attention, interest, and affect—their overall experience in engaging with the specific mHealth technology [10,12].

“Patient activation” is a patient-centered care outcome measure that refers to patients’ “willingness and ability to take independent actions to manage their health,” such as avoiding health-damaging behaviors and adopting healthy lifestyle choices, including exercising regularly, eating well, or monitoring their glucose levels [34]. DBCIs usually attempt to evaluate patient activation [34-36], although they may indicate that they are designed to assess adherence or engagement. “Adherence” is often confused with “engagement” and has been defined in at least three separate ways: adherence could refer to whether the intervention is used as intended by the developers [10,32], the usage of mHealth [37] (which is more accurately called engagement), or the patient’s willingness and ability to adhere to the recommendations provided by their physician or health care provider (which is likely the most common use of the word “adherence” in medico-legal parlance) [35,38-40].

Taken together, patient engagement typically refers to patients’ engagement with an intervention itself, whereas patient activation refers to patients’ physical and mental health-related activities based on what they learned from an intervention [34,41]. Patients can be engaged by, for example, reading digital messages on the importance of exercising and still not be activated to start exercising [34].

Patient engagement and activation are distinct outcome measures from patient experience in that patient experience refers to patients’ perceptions of others’ actions, whereas patient engagement and activation refer to patients’ actions [27,42]. Patient experience, engagement, and activation are all measures of patient-centered care in that they put emphasis on the patient playing an integral role in their outcomes [43,44], with the ultimate decision-making authority resting with patients. Further, all three concepts can be evaluated empirically.

Finally, “patient satisfaction” is the most attenuated outcome measure of patient-centered care. Patient satisfaction is a term that is often used in health care quality parlance and yet is frequently misunderstood [27] owing to one key feature of satisfaction that is often ignored: satisfaction has little to do with quality [22,27,29]. Patient satisfaction only refers to whether patients’ expectations were met [22,27]. Patients can be satisfied with care that is low quality and yet be dissatisfied with high-quality care. For this reason, it is incorrect to say that the Hospital Consumer Assessment of Healthcare Provider and Systems survey, the first standardized, publicly reported survey of patient perspectives [45], measures patient satisfaction when, in actuality, the survey uses patient experience as its primary measure for patient-centered care [27,28,46].

As the above discussion illustrates, to date, we have not achieved a shared understanding of important mHealth constructs, or how to conceptualize and operationalize them [10]. Thus, if the mHealth community is to continue to promote the use of patient-centered mHealth technologies (ie, mHealth technologies that are designed with the goal of promoting patient-centered care, as measured by patient experience, patient engagement, patient activation, or patient satisfaction), then we need precision in our terminology to extrapolate generalizable, transferable results [6,31].

## Methodologies Used to Empirically Assess Patient-Centered Outcomes

To validly measure a concept, there must be a tight linkage between the patient-centered care construct (ie, the outcome measure) and items developed for measurement [47]. Below, we describe several methodologies that can assess each patient-centered outcome.

### Patient Experience

There are several qualitative methodologies that are ideal for assessing patient experience. Focus groups, semistructured interviews, observational studies, patient journey mapping, and walk-throughs are all appropriate to assess patient experience [21,29,48,49]. Semistructured interviews (where the interviewer uses a structured guide to aid conversation) are most helpful to elicit patients’ experiences, as well as to elicit particular informational and decisional needs. For example, consider an mHealth technology that provides educational messages (through text and email) to prepare and inform patients about their upcoming surgery. Asking patients to reflect on whether they had gaps in knowledge after completion of the module (eg, gaps in knowledge about surgery lifestyle impacts, surgical risks/benefits, and technical aspects of the surgery, or recovery trajectories and activities) would help elicit patients’ informational needs [29].

To assess patient experience, researchers should aim to elicit both definitional components of patient experience: (a) patients’ subjective and objective assessments of what occurred, and (b) patients’ observations across the full sequence of time [28,45]. Thus, if only one touchpoint of care within an episode is evaluated—such as patient experiences using a digital check-in registration system during a scheduled surgery—then it likely cannot be said that patient experience was fully assessed. A more precise methodology would be one in which teams systematically evaluate patient experiences through all touchpoints using walk-throughs or patient journey mapping, starting from appointment scheduling, to the registration check-in, to the digital navigation system that shows patients how to get to a particular department, to the Smart TVs or Smart Boards within the patients’ hospital rooms, and all the way to the patients’ beside tablet-based and electronic health record solutions [50-52]. The goal of walk-throughs and journey mapping is to ask patients what they are feeling, seeing, and experiencing as they move from, say, the registration portal to a patient room [50].

### Patient Engagement

Short and colleagues [10] describe several methodologies that are appropriate to evaluate patient engagement. To assess patient engagement, researchers should aim to elicit both definitional components of patient engagement: the objective component, as the extent (eg, amount, frequency, duration, and depth) of usage of the mHealth technology, as well users’ subjective assessment in using the mHealth technology [13]. The objective component is likely best evaluated using quantitative measures such as the number of login attempts, the time spent on a technology, the time spent reading a particular message or conducting an e-module, or the amount of bidirectional communication between a patient and provider using an mHealth technology [11,13,53-58]. The subjective component of engagement can likely best be evaluated using qualitative methodologies such as semistructured interviews and focus groups, which tend to be most appropriate for teasing out themes, as well as users’ beliefs, narratives, and perceptions [23].

### Patient Activation

There is a heavy behavioral dimension to patient activation—what the patient does in response to the intervention in terms of his or her health-related activities—which can be assessed quantitatively [58,59]. Several studies that claim to measure patient engagement are arguably instead measuring patient activation [60-62]. Whether a patient had higher medication adherence [63,64], higher levels of physical exercise [9,65,66], or improved diabetes management as a result of the mHealth intervention [67,68] can be considered patient activation if the patient took healthy actions based on what he/she learned through the mHealth technology. These medical, clinically based health outcomes can be empirically derived using electronic medical records. There are also validated, reliable quantitative measures available to evaluate patient activation [58,59].

### Patient Satisfaction

Patient satisfaction is generally considered to be the most attenuated outcome measure for patient-centered mHealth technologies and thus can likely be assessed from a purely descriptive [69] quantitative point of view, devoid of any thematic nuancing that qualitative measures can afford. There are numerous quantitative measures available to evaluate patient satisfaction, although the validity and reliability of the instruments have been a point of debate [69-80].

Table 1 outlines a common pathway linking definitions with outcomes and methodologies. The table is not intended to be exhaustive but is instead designed to provide a robust set of patient-centered constructs, outcome measures, and methodologies. In this summary, we refrained from including strictly objective, physiological measures because the patient-centered constructs depend heavily on the subjective experience of patients, which physiological measures often cannot elicit. 

**Table 1:** Patient-centered mobile health (mHealth) technologies: outcome measures, methodologies, and definitions.

Outcome measures	Definitions	Methodologies			Example mHealth Technologies
		Qualitative	Quantitative		
Patient experience	Any process observable by patients, including subjective experiences (eg, pain control) and their objective experiences (eg, wait time). Must refer to the entire sequence in the care episode or full continuum of care—from the first to the last touchpoint.	Semistructured interviews Think aloud exercises Focus groups	Walk-throughs (eg, the Walk Through Tool [48]) Patient Journey Mapping Tool [49,50] Impact of Assistive Devices Scale (PIADS) [51]	Touchscreen kiosks for registration or check-in Digital navigation systems to help patients navigate the hospital Smart TVs in hospital rooms	
Patient engagement	The extent (eg, amount, frequency, duration, and depth) of usage of the mHealth technology, coupled with the user’s subjective assessment in using the mHealth technology.	Semistructured interviews Think aloud exercises Focus groups	Self-report questionnaires (eg, the eHealth Engagement Scale and the Digital Behavior Change Intervention Engagement Scale [52]) Usability and acceptability scales (eg, The mHealth App Usability Questionnaire [MAUQ] [53]) Social Networking Time Use Scale (SONTUS) [54] Facebook Intensity Scale (FBI Scale) [55] Media and Technology Usage and Attitudes Scale (MTUAS) [56] Chinese Internet Gaming Disorder Scale [57] Number of logins; time spent on mHealth; time spent reading a message; number of monitoring questions for which there was a response	Two-way bidirectional communication on a communication or education mobile platform Secure SMS text message/email/push notifications, self-scheduling, medical record access, patient-provider messaging, and bill pay	
Patient activation	Willingness and ability to take independent actions to manage their health	Focus groups, semistructured interviews	Patient Activation Measure (PAM) [58,59]	Digital behavior change interventions Patient biosensor monitoring devices (eg, glucose monitoring kits)	
Patient satisfaction	Whether a patient’s expectations were met.	Semistructured interviews	Short Assessment of Patient Satisfaction (SAPS) [60] Risser Patient Satisfaction Scale [61] Patient Satisfaction Questionnaire (PSQ) (multiple iterations) [62] Quebec User Evaluation of Satisfaction with Assistive Technology (QUEST) [63]	Patient-reported outcomes collection via mHealth	

## Recommendations

Above, we have described several definitional inconsistencies for key constructs in the mHealth literature. In an effort to achieve clarity, we teased apart several patient-centered care outcomes, and we outline methodologies appropriate to measure each of the patient-care outcomes (Table 1). In what follows, we provide recommendations for evaluating mHealth patient-centered technologies. Our recommendations relate to the patient-centered constructs in that we advocate for patients taking center stage in mHealth research and collaboration efforts to enhance patient experience, patient engagement, patient activation, and patient satisfaction. 

### Recommendation 1: Use Patients When Assessing Patient-Centered Care mHealth Technologies

Patient participation is a central tenet of ethically driven research and product development [81-83]. Unfortunately, much of mHealth research only engages patients in the beginning phases during agenda-setting and protocol development [83]. Systematic reviews have found that little research involves patients throughout the development, implementation, and modification phases of mHealth patient-centered technologies [83,84]. Reviews have also found that patient participation is often treated as a tokenistic measure, one in which patients’ feedback is used primarily as a means of “rubberstamping” to secure funding or to approve a previously chosen decision made by the research or design team, rather than using patients as principal drivers of decision-making [83,84].

The main reason that patient participation is integral to conducting ethically driven mHealth research and product development is because patients are unique [42,85]; they should not be viewed as the same type of customers found in other sectors [6]. For instance, customers of any other good or service have luxuries that patients may not have. Patients become consumers, arguably not by choice but rather by need. Patients’ preferences are largely unknown when the good or service is used, and they have limited channels of communication and limited control [42]. Patients likely experience physical or emotional impairments such as fears, grief, and anxiety while using the service [86]. All of these factors likely suggest that a particular patient’s informational and decision-making needs are different from those that they would have outside of a health care context [86-89]. Patient participation and active engagement are the classic tenets of task, user, representation, and functional analysis used to inform system designers and leadership that are contemplating implementing solutions to patient-centered problems [87-89].

### Recommendation 2: Create an International Collaboration to Enhance the Quality and Effectiveness of mHealth Technologies

mHealth technologies are the future for patient-centered care [3,74]. However, the extent to which mHealth technologies influence or impact patient-centered care is unclear [6], which is likely owing to the imprecision in definitions, methodological approaches, and disjointed interests of multiple stakeholders within health care organizations and industry [2].

We contend that some form of self-regulation or an internationally used assessment framework is needed to ensure that quality standards are met before wide-scale dissemination of any patient-centered mHealth technology [2]. The US Food and Drug Administration has taken a passive approach, explicitly applying its regulatory oversight to only those software functions that are medical devices and whose functionality could pose a risk to a patient safety if the device were to not function as intended [90]. The European Commission mHealth Green Paper does not give any recommendations [91]. The Health Technology Assessment Agency and its collaborative networks have discussed the importance of a collaborative approach, but, at the time of this writing, have failed to provide some form of a comprehensive evaluative framework [92,93].

When patient decision aids were being developed and implemented at a rapid pace without high-quality evidence, an international collaboration among researchers, practitioners, and stakeholders was instituted to enhance the quality and effectiveness of patient decision aids, called the International Patient Decision Aids (IPDAS) Collaboration [94]. The group established (and routinely revises) an evidence-informed framework, which outlines actions that patient decision-aid developers should take in terms of content-writing, development, implementation, and evaluation. The IPDAS Collaboration “grades” decision aids based on whether and to what degree the aids meet parameters, and the score can be used in marketing and evaluating the patient decision aid [94].

An international collaboration similar to that used for patient decision aids is, we believe, appropriate for the regulation and systematic evaluation of patient-centered mHealth technologies. Similar to patient decision aids, the successful implementation of patient-centered mHealth requires multidisciplinary teams from academia, industry, and health care management sectors, along with patients and consumers working collaboratively to maintain the requisite medical, statistical, information technology, patient-centered, and research expertise necessary to implement and evaluate mHealth technologies [95], which an international collaboration would afford. There are several actions a large collaboration could take to encourage high-quality development and dissemination of digital technologies.

First, because of its large scale, an international collaboration would be well-positioned to help address barriers to electronic interoperability issues that stem from disparate proprietary digital health record systems by, for example, creating digital health data exchange platforms to standardize data [95]. Second, a collaboration could disseminate practical advice on how organizations can use their foundational digital systems to leverage existing capabilities for achieving coordination through bolt-on, incremental development of digital technologies. Third, an international collaboration could build upon our work to develop exhaustive criteria and methodology standards for how to design, produce, implement, and evaluate digital technologies.

## Conclusion

In this Viewpoint, we called for interdisciplinary, conceptual clarity on the definitions, methodologies, and patient-centered outcomes frequently used in mHealth research. In doing so, we advocate for consideration of several measures of patient-centered care, and we outline various methods for evaluating them. By creating a common pathway linking definitions with outcomes and methodologies, we provide a possible interdisciplinary approach to evaluating mHealth technologies.

To that end of creating an interdisciplinary approach, we also provide several recommendations that we believe can advance mHealth research and implementation. For instance, if an international collaboration were created to develop evaluative criteria, using the guidance provided here to ground criteria development, then low-quality digital technologies would likely be excluded. Transparency and precision would be promoted, large-scale published evidence would be encouraged [3], and mHealth technologies could finally flourish within a high-quality, patient-centered landscape.

## Appendix

Multimedia Appendix 1 Data Abstraction Table.

## Abbreviations

DBCI: digital behavior change intervention
IOM: Institute of Medicine
IPDAS: International Patient Decision Aids
mHealth: mobile health
